# Fault Detection and Isolation of the Multi-Sensor Inertial System

**DOI:** 10.3390/mi12060593

**Published:** 2021-05-21

**Authors:** Hao Liang, Yu Guo, Xingfa Zhao

**Affiliations:** 1 School of Automation, Nanjing University of Science and Technology, Nanjing 210094, China; lianghao_china@126.com; 2 Beijing Aerospace Times Laser Inertial Technology Company, Ltd., Beijing 100094, China; xfzhao126@163.com

**Keywords:** the inertial measurement unit, fault detection, fault isolation, the generalized likelihood test method, grey model

## Abstract

In order to solve the problem that the generalized likelihood test method cannot isolate the single fault of the four-gyro system and the double faults of the six-gyro system, a fault detection and isolation method combining the generalized likelihood test method with the residual error of the metabolism grey model is presented. The problem of isolating the single fault of the four-gyro system and the double faults of the six-gyro system using the generalized likelihood test method is analyzed. The method and process of fault detection and isolation are designed. The validity of the method presented in this paper is verified by simulation tests of the single fault of the four-gyro system and the double faults of the six-gyro system. By comparing the isolation performance with the generalized likelihood test method, it is proved that the isolation performance of the method proposed in this paper is better than that of the generalized likelihood test method. The method mentioned in this paper can effectively realize fault detection and isolation of the multi-gyro system and improve the inertial system’s reliability.

## 1. Introduction

The inertial measurement unit [1,2,3] comprises at least three single-axis angular velocity sensors and three single-axis acceleration sensors. The angular velocity sensor and the acceleration sensor are used to measure the angular velocity and the acceleration of an object in three-dimensional space, respectively. The attitude of the object is calculated by measuring the angular velocity and acceleration in three-dimensional space. It has significant application value in navigation.

The strap-down inertial measurement unit is the core equipment of a navigation system in aeronautics, astronautics and warships. Not only its output accuracy is important to the carrier movement, but its reliability is also an essential factor to make sure whether the carrier can work properly. Any system composed of multiple sensors will face the problem of sensor failure, and the same is true for inertial equipment. In practice, due to the influence of the environment, component lifespan, design rationality and other aspects, the device’s internal sensors will inevitably fail. Therefore, it is significant to improve the reliability of the strap-down inertial measurement unit.

The inertial measurement unit needs at least three gyros to measure the three-dimensional angular velocity of the object. For the reliability of the system, ensuring that the number of effective working sensors is not less than three can make the system work normally. Therefore, redundant inertial measurement combined with multi-sensor configuration can significantly improve the reliability of the system [4,5,6]. When one or more sensors fail, fault detection and isolation methods are used to isolate the fault sensors, thus realizing the system’s reconfiguration, which can ensure that the system still works properly.

In the design of inertial equipment, the more redundant sensors do not mean the better. The reliability, volume and cost of the equipment need to be considered comprehensively. Generally speaking, when designing redundant strap-down inertial equipment, a four-gyro redundant system or a six-gyro redundant system is usually used according to a different emphasis. For the four-gyro system, its fault tolerance is one gyro. Additionally, for the six-gyro system, its fault tolerance is three gyros. Therefore, it is significant to study the single fault of the four-gyro system and the double faults of the six-gyro system.

Many researchers have studied the fault detection and isolation methods of redundant sensor inertial measurement systems. The most commonly used methods include the singular value decomposition method [7], the generalized likelihood test method [8], the optimal parity vector method [9,10,11], etc. Among these methods, the generalized likelihood test method has the advantages of high sensitivity, high real-time performance and less computation. However, it separates fault detection and fault isolation, which causes some limitations. Considering the cost and reliability, the four-gyro system and the six-gyro system are used in more multi-sensor inertial systems. The laser inertial unit of Japan’s H-2A rocket and the strap-down inertial navigation system of the EOS-AQUA satellite in the United States both use the four-gyro system. The fault-tolerant air data inertial reference system of Boeing 777 aircraft and the redundant inertial flight control assembly of the Delta II rocket launcher both use the six-gyro system. For a four-gyro system, the generalized likelihood test method can only detect system faults but cannot isolate the fault sensor. For a six-gyro system, the generalized likelihood test method cannot isolate the double faults. It is necessary to combine other fault isolation methods to isolate faults. Oliveira [5] used χ^2^-cusum and wavelet packets to realize fault detection and isolation of the four-gyro system. Cheng [12] used an improved generalized likelihood test and Kalman filter to realize fault detection and isolation of the six-gyro system. However, the two methods lack generality, and the technology of the two methods is more complex and lacks real-time. In this paper, two typical problems of the single fault of the four-gyro system and the double faults of the six-gyro system are studied. The method in this paper is also applicable to the multi-fault of a six-gyro system.

The grey system theory is a control theory of a system with incomplete or uncertain information. By processing, generating and utilizing the small sample information, the grey system theory can obtain the potential law of the system and realize the prediction. The grey series prediction uses a dynamic grey model to predict the system’s time series. Compared with other fitting and prediction methods, the grey method has no strong constraints on the data and can obtain higher fitting accuracy and simple calculation.

In this paper, a novel fault detection and isolation method based on a generalized likelihood test combined with the residual error of the metabolism grey model (RE-MGM) is proposed. According to the error variation between the predicted output and the actual output, the residual vector of the gyro is obtained and then combined with the generalized likelihood test method to realize the fault detection and isolation. The fault isolation method proposed in this paper can make up for the isolation failure of the generalized likelihood test method in the case of the single fault of the four-gyro system and the double faults of the six-gyro system. At the same time, the isolation accuracy of the single fault for the multi-gyro system is better than that of the generalized likelihood test method. The fault detection and isolation method proposed in this paper can effectively detect and isolate multi-fault of the multi-gyro system. The method proposed in this paper can be used for failure and system reconfiguration of the multi-sensor systems many times. For example, a six-gyro system has double faults and single fault successively.

The structure of this paper is as follows: Section 1 introduces the relevant background, the problems faced and the contributions of this paper. Section 2 introduces the theory of the generalized likelihood test method and the isolation problem of the generalized likelihood test method in a multi-sensor system. Section 3 designs a metabolism grey model to predict gyro output. Section 4 explains the proposed approach in detail. Section 5 examines the proposed approach and carries out a comparison with the generalized likelihood test method to validate the effectiveness and superiority. Section 6 summarizes the full text.

## 2. Fault Detection and Isolation Based on the Generalized Likelihood Test Method

### 2.1. The Theory of the Generalized Likelihood Test Method

Suppose that a strap-down inertial measurement system with *p* angular velocity sensors has *q* measurement axes, then the expression of the measurement equation is as follows [5]:

(1) M=HΩ+η+ε

where $M\in R^{p}$ is the measurement output vector of *p* sensors; $\Omega \in R^{q}$ is the input vector of the physical quantity to be measured; p≥q; $H\in R^{p\times q}$ is the installation matrix of the sensors of the measurement system; $\eta \in R^{p}$ is the fault vector. When a sensor fails, its corresponding member value is the fault eigenvalue, and the corresponding member value of the sensor that does not fail is 0; $\varepsilon \in R^{p}$ is the *p*-dimensional measurement noise vector of the sensor. Suppose the sensor measures noise that satisfies the Gaussian noise $N(0,\sigma^{2})$, that is, E(ε)=0, $E(\varepsilon \varepsilon^{T})=\sigma^{2}I_{p}$.

The decoupling matrix $V\in R^{(p-q)\times p}$ is introduced, and the parity vector $g\in R^{p-q}$ is obtained by a linear transformation of Equation (1). The transformation process is as follows [12]:

(2) g=VM=VHΩ+Vη+Vε

A constraint is added to make the parity vector *g* independent of the input vector. The decoupling matrix *V* satisfies

(3) $$VH=0,\;VV^{T}=I_{p-q}$$

Equation (2) changes to:

(4) g=Vη+Vε

As can be seen from Equation (4), the parity vector *g* is not related to the measurement vector but only to the fault vector η and the noise vector ε.

According to the constraints of the decoupling matrix *V* and the installation matrix *H*, it is known that the decoupling matrix *V* is in the left zero space of the matrix *H*. Potter [13] chooses the matrix *V* as an upper triangular matrix with positive diagonal elements and then obtains the elements of the decoupling matrix *V* by orthogonalization.

Two hypotheses about the parity vector g are defined, respectively. The case of no-fault is defined as *H*_0_, and the case of fault is defined as *H*_1_. Since the noise vector ε satisfies the Gaussian distribution, the statistical properties of the parity vector g can be obtained from Equation (4) as follows [12]:

(5) $$H_{0}:E(g)=0,D(g)=E(gg^{T})=\sigma^{2}VV^{T}$$

(6) $$H_{1}:E(g)=V\eta ,D(g)=E((g-V\eta ){\left(g-V\eta \right)}^{T})=\sigma^{2}VV^{T}$$

Since the parity vector g satisfies the *p*-dimensional normal distribution, the likelihood Equations (7) and (8) under two hypotheses can be obtained.

(7) $$L\left(g|H_{0}\right)=\frac{1}{{\left\|\sigma^{2}VV^{T}\right\|}^{1/2}{\left(2\pi \right)}^{(p-q)/2}}\exp\left\{-\frac{1}{2}g^{T}\frac{1}{\sigma^{2}VV^{T}}g\right\}$$

(8) $$L\left(g|H_{1}\right)=\frac{1}{{\left\|\sigma^{2}VV^{T}\right\|}^{1/2}{\left(2\pi \right)}^{(p-q)/2}}\exp\left\{-\frac{1}{2}{\left(g-V\eta \right)}^{T}\frac{1}{\sigma^{2}VV^{T}}(g-V\eta )\right\}$$

The logarithm likelihood ratio Equation (9) can be obtained from Equations (7) and (8):

(9) $$L(g)=\ln\left[\frac{L\left(g|H_{1}\right)}{L\left(g|H_{0}\right)}\right]=\frac{1}{2}\left[g^{T}\frac{1}{\sigma^{2}VV^{T}}g-{\left(g-V\eta \right)}^{T}\frac{1}{\sigma^{2}VV^{T}}(g-V\eta )\right]$$

The maximum likelihood Equation (10) can be obtained from Equation (9).

(10) $$L_{\max}(g)=\frac{1}{2\sigma^{2}VV^{T}}\left[g^{T}g\right]$$

Therefore, the following fault detection function is constructed using *g*:

(11) $$D_{GLT}=\frac{1}{\sigma^{2}}\left[g^{T}g\right]=\frac{1}{\sigma^{2}}\sum_{i=1}^{p-q}g_{i}^{2}$$

where $D_{GLT}\sim \chi^{2}(p-q)$. The detection threshold $T_{D}$ is set by the number of gyros and the false alarm rate α. When $D_{GLT}\geq T_{D}$, a fault is detected.

When the fault is detected, it is necessary to isolate the fault sensor. The failure of the *i*th sensor is defined as *H_i_*, and its logarithm likelihood equation is as follows:

(12) $$\ln\left[L\left(g|H_{i}\right)\right]=\ln\frac{1}{{\left\|\sigma^{2}VV^{T}\right\|}^{1/2}{\left(2\pi \right)}^{(p-q)/2}}-\frac{1}{2}{\left(g-v_{i}\eta_{i}\right)}^{T}\frac{\left(g-v_{i}\eta_{i}\right)}{\sigma^{2}VV^{T}}$$

The maximum likelihood estimation of Equation (12) can be obtained.

(13) $$\ln{\left[L\left(g|H_{i}\right)\right]}_{\max}=\ln\frac{1}{{\left\|\sigma^{2}VV^{T}\right\|}^{1/2}{\left(2\pi \right)}^{(p-q)/2}}-\frac{1}{2}\frac{g^{T}g}{\sigma^{2}I_{p-q}^{}}+\frac{1}{2}\frac{{\left(g^{T}v_{i}\right)}^{2}}{\sigma^{2}VV^{T}v_{i}^{T}v_{i}}$$

Therefore, when the *i*th sensor fails, the following fault isolation function is defined:

(14) $$I_{GLT}(i)=\frac{{\left(g^{T}v_{i}\right)}^{2}}{v_{i}^{T}v_{i}}$$

It can be seen from Equations (13) and (14) that the larger the value of the fault isolation function is, the larger the value of the corresponding maximum likelihood function is, indicating that the corresponding sensor has a greater probability of failure. Therefore, each sensors’ value of fault isolation function is calculated, and the sensor corresponding to the maximum value is the faulty sensor.

### 2.2. Isolation Problem of the Multi-Sensor System

#### 2.2.1. The Four-Gyro System

The generalized likelihood test method is used to detect the fault of the four-gyro system. It is necessary to identify the fault sensor for further isolation and system reconstruction when the fault is detected. For the four-gyro system, if the generalized likelihood test method is used for fault isolation, it can be seen from Equation (14) that the isolation function of the four-gyro system is:

(15) $$I_{GLT}(i)=\frac{{\left(g^{T}v_{i}\right)}^{2}}{v_{i}^{T}v_{i}},i=1,2,3,4$$

According to Equation (2), for the four-gyro system, the parity vector’s dimension is 4 − 3 = 1, so it is scalar. The parity matrix $V\in R^{(p-q)\times p}$’s dimension is 1×4, so it is also a scalar for each column $v_{i}$.

Therefore, Equation (15) can be calculated as:

(16) $$I_{GLT}(i)=\frac{{\left(g^{T}v_{i}\right)}^{2}}{v_{i}^{T}v_{i}}=\frac{{\left(gv_{i}\right)}^{2}}{v_{i}^{}v_{i}}=\frac{g^{2}v_{i}{}^{2}}{v_{i}^{2}}=g^{2}$$

It can be seen from Equation (16) that, for the four-gyro system, the fault isolation function of each sensor is the same value, in which case the fault gyro cannot be separated when the fault is detected. Therefore, another method must be used in combination with the generalized likelihood test method for fault isolation.

#### 2.2.2. The Six-Gyro System

Using the generalized likelihood test method, in n-dimensional space, (*n* + 1) gyros can only be detected fault but not isolated fault [14]. If the system can separate single fault of *p* gyros, at least (*p* + *n* + 1) gyros need to be configured. If the system wants to separate *k* gyros with simultaneous failures, at least (2*k*+*n*) gyros need to be configured [14]. Thus, at least seven gyros are required to isolate double faults in three-dimensional space. For a six-gyro system, the generalized likelihood test method can only detect and isolate the single fault.

## 3. The Output Prediction of the Gyro Based on the Grey Model

### 3.1. The Theory of the Grey Model

The grey model [15] is an important grey dynamic prediction model in grey system theory. Its idea is to use a differential equation to model variables. It can fit and predict the characteristic data of a complex system.

A new sequence $x^{\left(1\right)}=\{x^{\left(1\right)}(1),x^{\left(1\right)}(2),\cdot \cdot \cdot ,x^{\left(1\right)}(n)\}$ can be obtained by using the first-order accumulated generating operation to the original sequence $x^{\left(0\right)}=\{x^{\left(0\right)}(1),x^{\left(0\right)}(2),\cdot \cdot \cdot ,x^{\left(0\right)}(n)\}$, where $x^{\left(1\right)}(k)=\sum_{i=1}^{k}x^{\left(0\right)}(i),k=1,2,\cdot \cdot \cdot ,n$ [16].

Average every two adjacent data in $x^{\left(1\right)}=\{x^{\left(1\right)}(1),x^{\left(1\right)}(2),\cdot \cdot \cdot ,x^{\left(1\right)}(n)\}$ to obtain the mean sequence $z^{\left(1\right)}=\{z^{\left(1\right)}(2),z^{\left(1\right)}(3),\cdot \cdot \cdot ,z^{\left(1\right)}(n)\}$, where $z^{\left(1\right)}(k)=[x^{\left(1\right)}(k-1)+x^{\left(1\right)}(k)]/2,k=2,3,\cdot \cdot \cdot ,n$.

The grey difference equation of the GM(1,1) is expressed as:

(17) $$x^{\left(0\right)}(k+1)=a\left[-\frac{1}{2}\left(x^{\left(1\right)}(k)+x^{\left(1\right)}(k+1)\right)\right]+b$$

The whitenization equation is expressed as:

(18) $$\frac{dx^{\left(1\right)}}{dt}+ax^{\left(1\right)}=b$$

where *a* and *b* is the parameter to be determined, which is called developing coefficient and grey actuating quantity, respectively. $u={\left[a,b\right]}^{T}$ is the parameter vector to be identified. Define:

(19) $$Y=\left[\begin{array}{c} x^{\left(0\right)}(2)\\ x^{\left(0\right)}(3)\\ \vdots \\ x^{\left(0\right)}(n) \end{array}\right],\;B=\left[\begin{array}{cc} -z^{\left(1\right)}(2) & 1\\ -z^{\left(1\right)}(3) & 1\\ \vdots & \vdots \\ -z^{\left(1\right)}(n) & 1 \end{array}\right]$$

Then Equation (17) can be written as follows:

(20) Y=Bu

The least square method is used to estimate $u={\left[a,b\right]}^{T}$:

(21) $$\hat{u}={\left[\hat{a},\hat{b}\right]}^{T}={\left(B^{T}B\right)}^{-1}B^{T}Y$$

By bringing the estimated value from Equation (21) into Equation (18), the following analytical solutions can be obtained:

(22) $${\hat{x}}^{\left(1\right)}(k+1)=\left[x^{\left(1\right)}(1)-\frac{\hat{b}}{\hat{a}}\right]e^{-\hat{a}k}+\frac{\hat{b}}{\hat{a}}$$

By using the first-order inverse accumulated generating operation to ${\hat{x}}^{\left(1\right)}(k+1)$, the predicted values of the original data are obtained:

(23) $$\begin{array}{ll} {\hat{x}}^{\left(0\right)}(k+1) & ={\hat{x}}^{\left(1\right)}(k+1)-{\hat{x}}^{\left(1\right)}(k)\\ & =\left(1-e^{\hat{a}}\right)\left[x^{\left(1\right)}(1)-\frac{\hat{b}}{\hat{a}}\right]e^{-\hat{a}k} \end{array}$$

Equations (22) and (23) are the calculation equations of the GM(1,1).

### 3.2. Output Prediction Based on the Metabolism GM(1,1)

The modeling process of the metabolism GM(1,1) can be regarded as using a sliding window to model and predict of the GM(1,1) [17].

Assuming that the number of window data is *n*, the sequence of a window in the original sequence is $x_{i}^{\left(0\right)}$, and the expression of $x_{i}^{\left(0\right)}$ is

(24) $$x_{i}^{\left(0\right)}=\{x^{\left(0\right)}(i+1),x^{\left(0\right)}(i+2),\cdot \cdot \cdot ,x^{\left(0\right)}(i+n)\}$$

According to the sequence $x_{i}^{\left(0\right)}$, the predicted value ${\hat{x}}^{\left(0\right)}(i+n+1)$ of the next data can be obtained. After getting the next real data, slide the window to obtain the next window sequence $x_{i+1}^{\left(0\right)}$, then the expression of $x_{i+1}^{\left(0\right)}$ is

(25) $$x_{i+1}^{\left(0\right)}=\{x^{\left(0\right)}((i+1)+1),x^{\left(0\right)}((i+1)+2),\cdot \cdot \cdot ,x^{\left(0\right)}((i+1)+n)\}$$

At this time, the next prediction data ${\hat{x}}^{\left(0\right)}((i+1)+n+1)$ can be obtained. By analogy, the predicted value ${\hat{x}}^{\left(0\right)}(t)$ can be obtained from the sequence:

(26) $$x_{t-n-1}^{\left(0\right)}=\{x^{\left(0\right)}(t-n),\cdot \cdot \cdot ,x^{\left(0\right)}(t-2),x^{\left(0\right)}(t-1)\}$$

from which a real-time predicted sequence can be obtained.

**Figure 1:**
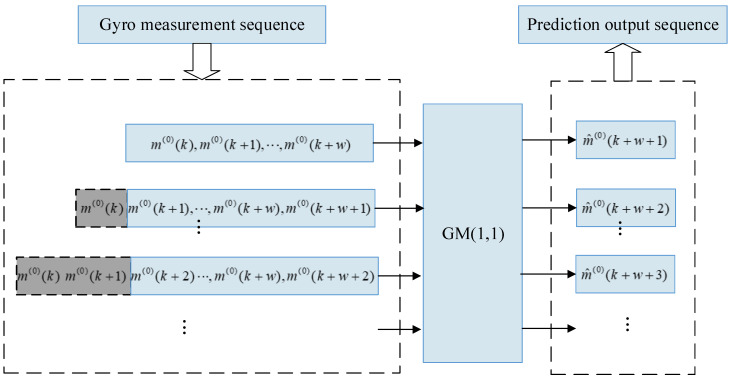
Structure chart of the Gyro’s predictive output based on the metabolism GM(1,1).

As is presented in Figure 1, the gyro’s measurement sequence enters the input window of the model in turn as the input sequence of the GM(1,1). The GM(1,1) is used to generate prediction for the next moment data, and then the measurement sequence inputs new measurement data into the window, removing the oldest measurement data. By analogy, a gyro prediction sequence is generated. Since the GM(1,1) requires input to be non-negative, if all input sequences are negative, it is only necessary to reverse the input sequence to be positive and the prediction sequence to be negative. If the sequence contains positive and negative values, a constant offset can be superimposed on the input sequence and subtracted from the prediction sequence. Only positive input sequences are considered in this paper.

The gyro continuously updates the output of new data, reflecting the data’s characteristics at the next moment, so using new data can predict the following output data. Simultaneously, the old data information is removed in time so that the data modelling sequence can better reflect real-time characteristics of the gyro output. On the other hand, because the calculation and memory of the computer are limited, predicting with the data update of metabolism constantly can avoid the problem that the computer’s memory consumption increases with the increase of data which leads to the increasing amount of modelling operation. It can satisfy the real-time modelling of gyro data and obtain the real-time prediction of the gyro.

## 4. Fault Detection and Isolation Method Combined with the RE-MGM

When the gyro is working usually, due to the continuity of the carrier motion, when the sampling rate is high, the measurement output sequence of the gyro will not change abruptly but should be a stable change process, and the predicted data will also change steadily. Therefore, the error between the predicted and measured values should be small and stable. When the gyro fails, there will be an apparent step change in its measurement output pulse, which will result in a significant jump in error between its predicted value and the measured value. At this time, the error of other non-faulted gyros should remain stable.

The error between the predicted data obtained by the metabolism GM(1,1) and the actual sample data is defined as

(27) $$e(t)=x^{\left(0\right)}(t)-{\hat{x}}^{\left(0\right)}(t)$$

Set the data length of the fault isolation detection window to ξ and introduce the window isolation error matrix $E\in R^{p\times \xi }$, which is defined as

(28) $$E=[E_{1}\;E_{2}\;\ldots \;E_{\xi }]=\left[\begin{array}{cccc} e_{1(t-\xi +1)} & e_{1(t-\xi +2)} & \ldots & e_{1(t)}\\ e_{2(t-\xi +1)} & e_{2(t-\xi +2)} & \ldots & e_{2(t)}\\ \ldots & \ldots & \ldots & \ldots \\ e_{p(t-\xi +1)} & e_{p(t-\xi +2)} & \ldots & e_{p(t)} \end{array}\right]$$

where the matrix elements are calculated by Equation (27).

The measured data is noisy and fluctuating, and the data predicted by the metabolism GM(1,1) will also fluctuate. In order to reduce the error fluctuation caused by data noise interference and place the residual near zero, the mean error vector $\bar{E}\in R^{p}$ is introduced, which is defined as

(29) $$\bar{E}=\frac{1}{\xi -1}\sum_{j=1}^{\xi -1}\left|E_{j}\right|$$

The residual vector $\hat{E}\in R^{p}$ of the current moment is defined as

(30) $$\hat{E}=E_{\xi }-\bar{E}=\left[\begin{array}{c} e_{1(t)}-\frac{1}{\xi -1}\sum_{j=1}^{\xi -1}\left|e_{1(t-\xi +j)}\right|\\ e_{2(t)}-\frac{1}{\xi -1}\sum_{j=1}^{\xi -1}\left|e_{2(t-\xi +j)}\right|\\ \ldots \\ e_{p(t)}-\frac{1}{\xi -1}\sum_{j=1}^{\xi -1}\left|e_{p(t-\xi +j)}\right| \end{array}\right]$$

In the residual vector, the values corresponding to fault-free gyros should be distributed near zero, and the values corresponding to fault gyros will deviate significantly from zero. The residual vectors’ elements are sorted in descending order, and the gyro corresponding to the first element in the order is the gyro with fault.

**Figure 2:**
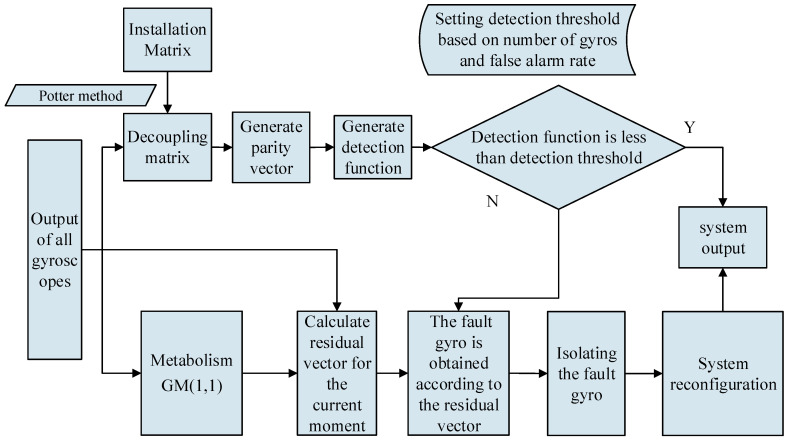
Structure diagram of fault detection and isolation based on the RE-MGM and the generalized likelihood test method.

Figure 2 illustrates the process of fault detection and isolation of the multi-gyro system using the generalized likelihood test method and RE-MGM. The system obtains the decoupling matrix *V* through the Potter method according to the installation matrix *H*. According to Equation (4), the parity vector g of the measurement output vector at the current time is obtained. Then, the value of the fault detection function at the current time is calculated according to Equation (11). Simultaneously, the gyro output generates the prediction sequence through the metabolism GM(1,1) and calculates the residual vector. The system calculates the detection threshold according to the number of gyros and the false alarm rate and compares it with the fault detection function’s value. If there is no fault, the system will output normally. If the fault is judged, the fault gyro is judged according to the residual vector, and the system is reconstructed. Then, send the reconstructed output.

## 5. Testing and Analysis

### 5.1. Verification with Four-Gyro System

A four-gyro system with three orthogonal gyros and one oblique gyro is simulated, and its installation matrix is configured as

(31) $$H=\left[\begin{array}{ccc} 1 & 0 & 0\\ 0 & 1 & 0\\ 0 & 0 & 1\\ 0.5774 & 0.5774 & 0.5774 \end{array}\right]$$

The simulation parameters are set as follows:

(1) All gyros are independent, and the noise satisfies the Gaussian distribution. The variance σ=0.01 can be used to fit most gyros. The false alarm rate is related to fault detection but not to fault isolation and the false alarm rate α=0.001.

(2) The simulation time is 5 s. The increase of the sampling rate can make the changing trend of the sequence more stable. 200 Hz sampling rate is commonly used in the inertial system, so the sampling rate is set to 200 Hz, and there are 1000 sampling points. The fault is injected at the 501st sampling point. The detection window ξ=5.

(3) The lower the angular velocity is, the more stable the trend of the sampling sequence is. In order to verify the performance at a higher angular velocity, the tri-axis angular velocity is set to 200°/s.

(4) The fault amplitude is 5σ and 10σ, respectively, and each gyro is injected with faults in turn.

**Figure 3:**
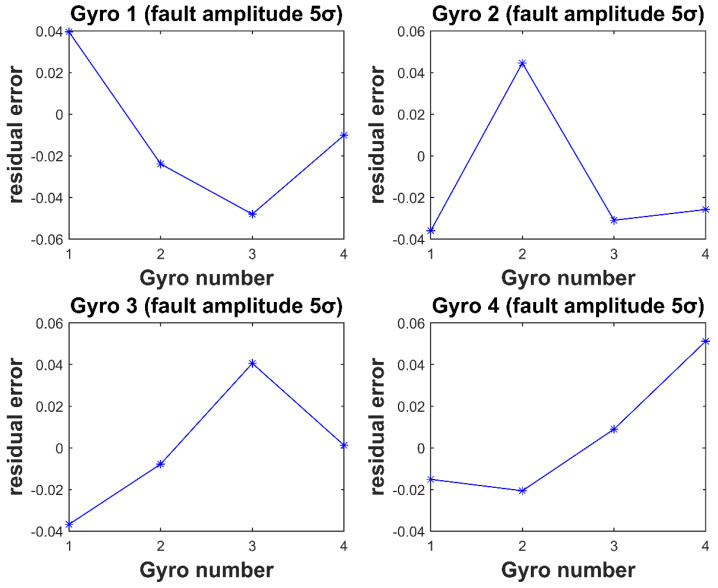
Residual vector values for Gyro 1–4 injection of 5σ fault amplitude.

**Figure 4:**
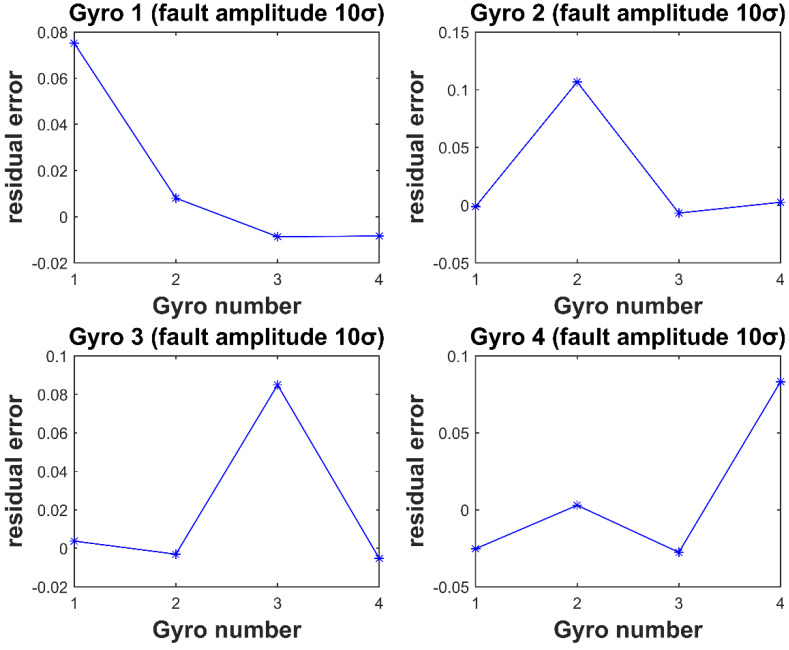
Residual vector values for Gyro 1–4 injection of 10σ fault amplitude.

In Figure 3 and Figure 4, the horizontal coordinate is the gyro number, and the vertical coordinate is the element value corresponding to each gyro fault residual vector at the time of the fault. It can be seen that for each gyro injected with fault, the element value corresponding to the fault isolation vector is the largest. Thus, isolating the fault gyro can be achieved.

### 5.2. Verification with the Six-Gyro System

#### 5.2.1. Single Fault Test

To verify the generality of this method, the four-gyro system is extended to a six-gyro system. For the six-gyro system, the number of rows and columns of the window isolation error matrix *E* is 6×ξ, and the dimensions of both the mean error vector $\bar{E}$ and the residual vector $\hat{E}$ are six. The six gyros of the system are symmetrically mounted along the normal direction of the dodecahedron with the following installation matrix:

(32) $$H=\left[\begin{array}{ccc} 0.5257 & 0 & 0.8507\\ -0.5257 & 0 & 0.8507\\ \;0.8507 & 0.5257 & 0\\ \;0.8507 & -0.5257 & 0\\ 0 & 0.8507 & 0.5257\\ 0 & 0.8507 & -0.5257 \end{array}\right]$$

**Figure 5:**
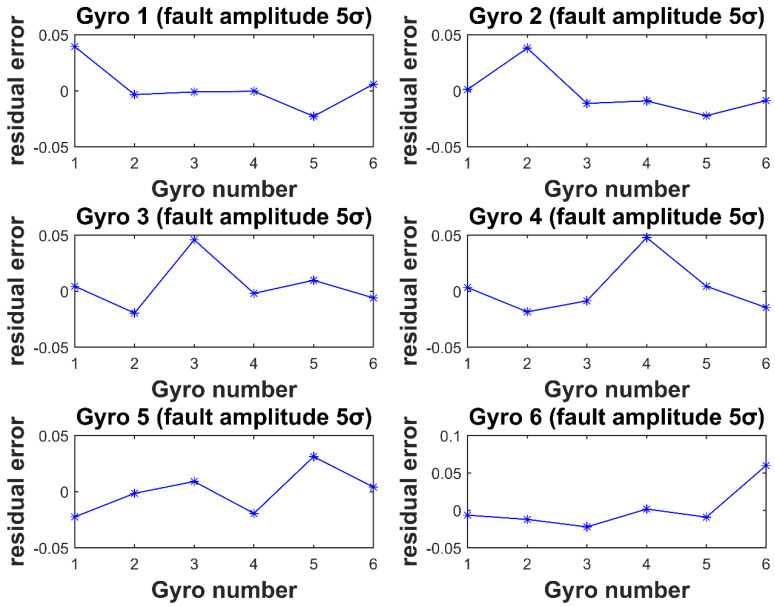
Residual vector values for Gyro 1–6 injection of 5σ fault amplitude.

**Figure 6:**
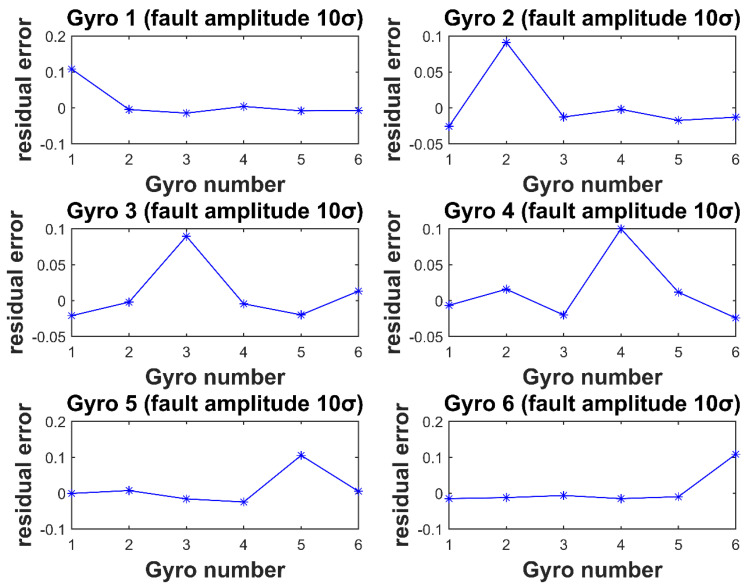
Residual vector values for Gyro 1–6 injection of 10σ fault amplitude.

The simulation parameters are consistent with the four-gyro system. Inject fault one by one to six gyros of the system. As shown in Figure 5 and Figure 6, injecting fault to six gyros, respectively. For the gyro with injection fault, the corresponding element value of the residual vectors is the largest. Thus, the fault gyro can be isolated.

#### 5.2.2. Double Faults Test

**Figure 7:**
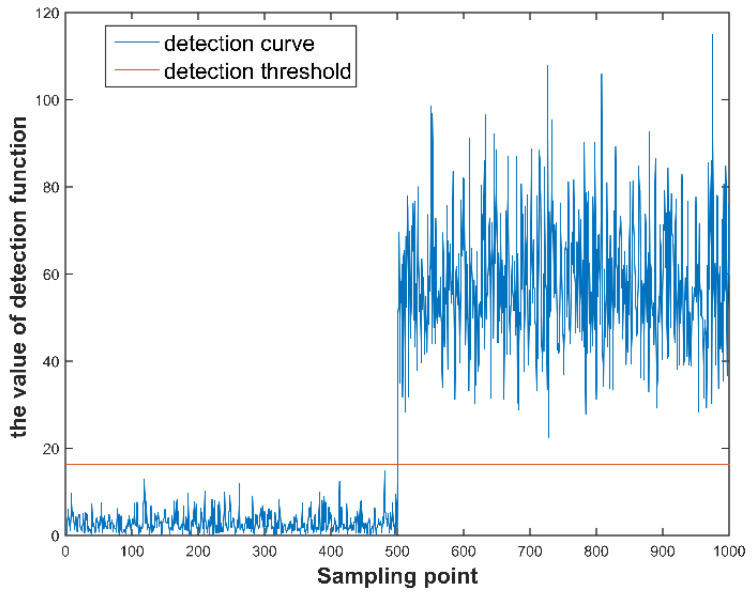
Fault detection curve for double faults.

**Figure 8:**
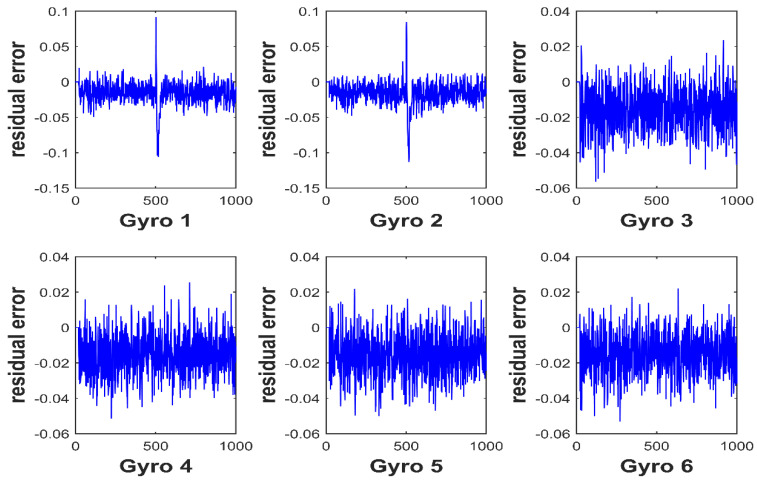
Residual curve for double faults.

Gyro 1 and gyro 2 are injected with 10σ fault at the same time. As shown in Figure 7, the value of the generalized likelihood test method’s fault detection function is greater than the detection threshold at the time of the fault occurrence. As shown in Figure 8, the residual curves of the first and second gyros jump obviously at the time of the fault occurrence. The corresponding residual values are larger than other gyros. Therefore, it can be identified that gyro 1 and gyro 2 are faulty.

The simulation and analysis of the four-gyro system and the six-gyro system show that the fault isolation method proposed in this paper can solve the problem that the four-gyro system cannot be isolated by the generalized likelihood test method. Simultaneously, it can be extended to the six-gyro system to isolate single and double faults, which proves the universality of the method to some extent.

### 5.3. Comparison of Fault Isolation Performance

Since the generalized likelihood test method cannot isolate the four-gyro system, the six-gyro system is used to compare the generalized likelihood test method’s performance with the RE-MGM method. The simulation parameters are the same as before, the fault range is set to 3σ–15σ, and the number of tests is 100.

As shown in Table 1, six gyros are injected with different amplitudes of faults in turn, and two methods are used to isolate the faults of six gyros. If the gyro with the injection fault can be isolated, the isolation is correct. By counting the correct isolation rate of 100 tests, the following conclusions can be drawn:

(1) Both methods increase the isolation accuracy with the increase of fault amplitude. The measured noise follows Gaussian distribution, and most of the noise values are within ±3σ, but there is also a probability that a larger noise value will be generated. In the case of small-amplitude faults, faults are easily drowned by noise, which affects the isolation function and the isolation accuracy rate. However, when the fault amplitude is large, the influence of noise is small, and the isolation accuracy rate is high. Above a certain fault amplitude, the average isolation accuracy rate can reach 100%.

(2) The method proposed in the paper isolation performance is better than the generalized likelihood test method. It can be seen from the average accuracy rate that when the fault amplitude is 3σ, the average fault isolation accuracy rate can reach 87.2%, which is better than 58.5% by using the generalized likelihood test method. Meanwhile, when the fault amplitude is 9σ, the generalized likelihood test method’s average fault isolation accuracy reaches 100%. In comparison, the method proposed in the paper reaches 100% when the fault amplitude is 6σ.

## 6. Conclusions

In this paper, a fault isolation method based on the residual error of the metabolism GM(1,1) is proposed. Combined with the generalized likelihood test detection method, the fault detection and isolation of the multi-sensor system can be achieved, and the inertial system’s reliability can be further improved.

Through the simulation test of the single fault of the four-gyro system and the double faults of the six-gyro system, and the comparison test of the isolation performance of the proposed method and the generalized likelihood test method in the single fault of six-gyro system, it is proven that this method can effectively compensate for the deficiency of the generalized likelihood test method and realize the detection of the single fault of the four-gyro system and the double faults of the six-gyro system. Compared with the generalized likelihood test method, the average isolation accuracy of the method proposed in the paper is improved by 49% at 3σ and 26% at 4σ. In addition, the average isolation accuracy is 100% at 6σ, which is better than 9σ using the generalized likelihood test method. The test proved that its isolation performance is better than the generalized likelihood test method.

The method proposed in this paper is to use the gyro output sequence directly and requires fewer samples. The calculation is simple, and the real-time update is excellent. Simultaneously, the method proposed in this paper is suitable for the multi-fault isolation of multi-gyro systems and has a wide range of applications. The method proposed in this paper has great application value in improving the reliability of redundant inertial systems, which can be widely used in multi-sensor systems.

The contributions of this paper are as follows:

(1) In this paper, a fault isolation method is proposed, which can solve the problem that the generalized likelihood test method cannot isolate the single fault of the four-gyro system.

(2) The proposed method can effectively make up for the defect that the generalized likelihood test method cannot isolate multi-fault of the multi-gyro system.

(3) In the case of the single fault of the multi-gyro system, the fault isolation accuracy of the proposed method is higher than that of the generalized likelihood test method.

(4) In this paper, a fault detection and isolation method combined with the generalized likelihood test method is proposed, which can effectively detect and isolate the single fault and the multi-fault of the multi-gyro system.

The research on fault detection and isolation of the multi-sensor system has great engineering application value in the inertial navigation system. The future research work includes: (1) Combined with other fault detection methods, we will study the feasibility of the method presented in this paper. (2) The fault isolation method based on the high-order grey model will be studied. (3) Combined with the grey theory, we will study how to further reduce the false alarm rate of the generalized likelihood test method.

## Figures and Tables

**Table 1:** Comparison of fault isolation accuracy rate between two methods.

Fault Amplitude (Times of σ)	The Fault Isolation Accuracy Using Generalized Likelihood Test Method (%)							The Fault Isolation Accuracy Using the RE-MGM Method (%)						
	The Gyro Number						Average Accuracy Rate	The Gyro Number						Average Accuracy Rate
	1	2	3	4	5	6		1	2	3	4	5	6	
3	59	57	59	59	58	59	58.5	83	87	91	85	87	90	87.2
4	75	77	75	76	76	75	75.7	97	96	98	95	93	95	95.7
5	88	86	89	88	87	89	87.8	100	100	99	100	100	99	99.7
6	94	94	95	94	94	96	94.5	100	100	100	100	100	100	100
7	98	99	99	98	98	97	98.2	100	100	100	100	100	100	100
8	99	100	99	100	99	99	99.3	100	100	100	100	100	100	100
9	100	100	100	100	100	100	100	100	100	100	100	100	100	100
10	100	100	100	100	100	100	100	100	100	100	100	100	100	100
11	100	100	100	100	100	100	100	100	100	100	100	100	100	100
12	100	100	100	100	100	100	100	100	100	100	100	100	100	100
13	100	100	100	100	100	100	100	100	100	100	100	100	100	100
14	100	100	100	100	100	100	100	100	100	100	100	100	100	100
15	100	100	100	100	100	100	100	100	100	100	100	100	100	100
